# Poor maternal nutrition and accelerated postnatal growth induces an accelerated aging phenotype and oxidative stress in skeletal muscle of male rats

**DOI:** 10.1242/dmm.026591

**Published:** 2016-10-01

**Authors:** Jane L. Tarry-Adkins, Denise S. Fernandez-Twinn, Jian Hua Chen, Iain P. Hargreaves, Viruna Neergheen, Catherine E. Aiken, Susan E. Ozanne

**Affiliations:** 1 Universityof Cambridge Metabolic Research Laboratories andMRC Metabolic Diseases Unit, Wellcome Trust-MRC Institute of Metabolic Science, Addenbrooke's Treatment Centre, Addenbrooke's Hospital, Hills Road, Cambridge CB2 OQQ, UK; 2 Neurometabolic Unit, National Hospital, University College London, London WC1N 3BG, UK

**Keywords:** Skeletal muscle, Oxidative stress, Mitochondria, Developmental programming

## Abstract

‘Developmental programming’, which occurs as a consequence of suboptimal *in utero* and early environments, can be associated with metabolic dysfunction in later life, including an increased incidence of cardiovascular disease and type 2 diabetes, and predisposition of older men to sarcopenia. However, the molecular mechanisms underpinning these associations are poorly understood. Many conditions associated with developmental programming are also known to be associated with the aging process. We therefore utilized our well-established rat model of low birth weight and accelerated postnatal catch-up growth (termed ‘recuperated’) in this study to establish the effects of suboptimal maternal nutrition on age-associated factors in skeletal muscle. We demonstrated accelerated telomere shortening (a robust marker of cellular aging) as evidenced by a reduced frequency of long telomeres (48.5-8.6 kb) and an increased frequency of short telomeres (4.2-1.3 kb) in vastus lateralis muscle from aged recuperated offspring compared to controls. This was associated with increased protein expression of the DNA-damage-repair marker 8-oxoguanine-glycosylase (OGG1) in recuperated offspring. Recuperated animals also demonstrated an oxidative stress phenotype, with decreased citrate synthase activity, increased electron-transport-complex activities of complex I, complex II-III and complex IV (all markers of functional mitochondria), and increased xanthine oxidase (XO), p67^phox^ and nuclear-factor kappa-light-chain-enhancer of activated B-cells (NF-κB). Recuperated offspring also demonstrated increased antioxidant defense capacity, with increased protein expression of manganese superoxide dismutase (MnSOD), copper-zinc superoxide dismutase (CuZnSOD), catalase and heme oxygenase-1 (HO1), all of which are known targets of NF-κB and can be upregulated as a consequence of oxidative stress. Recuperated offspring also had a pro-inflammatory phenotype, as evidenced by increased tumor necrosis factor-α (TNFα) and interleukin-1β (IL1β) protein levels. Taken together, we demonstrate, for the first time to our knowledge, an accelerated aging phenotype in skeletal muscle in the context of developmental programming. These findings may pave the way for suitable interventions in at-risk populations.

## Abstract (precis)

**Summary:** Muscle of ‘developmentally programmed’ rat offspring demonstrated accelerated aging and oxidative stress, which could explain why some individuals are at greater risk of developing age-associated muscular dysfunction than others.

## INTRODUCTION

For over 25 years, it has been known that a suboptimal *in utero* environment is strongly associated with increased risk of development of age-associated disease in later life, including cardiovascular disease (CVD) (Barker et al., 1989) and type 2 diabetes (T2D) (Barker et al., 1993). These findings have been robustly confirmed in both humans and animals (Tarry-Adkins and Ozanne, 2014; Zambrano et al., 2016), and these studies support the ‘thrifty phenotype hypothesis’ (Hales and Barker, 1992), which states that, under conditions of suboptimal nutrition, the fetus permanently alters its organ structure, metabolism and function to ensure immediate survival of the organism. Although beneficial in continued conditions of poor postnatal nutrition, such ‘developmental programming’ is known to be detrimental in postnatal conditions of adequate or over-nutrition, both of which can cause accelerated postnatal growth.

The development of skeletal muscle is especially vulnerable to nutritional deficiency compared to other tissues, owing to muscle mass being lost at the expense of brain-sparing *in utero* (Desai et al., 1996). Indeed, maternal nutrient restriction, a widely used model of developmental programming, is known to reduce offspring birth weight due to reductions in fetal circulating amino acids (Jansson et al., 2006; Pantham et al., 2015). This is highlighted in studies of ovine fetuses (Zhu et al., 2004) and offspring (Zhu et al., 2006) exposed to a suboptimal *in utero* environment, which demonstrate low birth weight as well as dysregulation of muscle development, including changes in the number and composition of myofibers. Numbers of myofibers and neuromuscular junctions are also altered in a rat model of maternal protein restriction (Confortim et al., 2016) and this effect is long-lasting, into old age (Confortim et al., 2015). There is also evidence that a suboptimal early environment (nutrient restriction) in the mouse can impact on muscle metabolism/function and molecular changes, including decreased mitochondrial content (Beauchamp et al., 2015) and reduced expression of mitochondrial genes, especially those involved in oxidative phosphorylation (Mortensen et al., 2010). Decreased muscle fiber score has also been observed in vastus lateralis muscle of low-birth-weight elderly men (Patel et al., 2012).

Muscle mass is known to decline with age, which can contribute to age-associated muscular dysfunction; however, the rate of decline shows great inter-individual variation (Sayer et al., 2010). With age, skeletal muscle can accumulate oxidative stress, which can cause issues such as a reduction in force generation and muscle atrophy. Muscle atrophy contributes to progressive weakness and an increased risk of mobility impairment, falls and physical frailty in very advanced age (Cruz-Jentoft et al., 2010). Among the most frequently implicated mechanisms of aging muscle atrophy is mitochondrial dysfunction, which leads to increased reactive oxygen species (ROS) generation (Marzetti et al., 2013).

Oxidative stress accumulation occurs when cellular ROS overwhelm the endogenous antioxidant defense capacity and thus redox homeostasis is not maintained (Ray et al., 2012). This excess ROS generation can cause macromolecular damage to proteins, lipids and DNA (Valko et al., 2007). Telomeres (hexamer repeats of DNA: [TTAGGG]*_n_*), which are found at the ends of chromosomes, are particularly susceptible to ROS damage because of their guanine-rich sequences (Oikawa and Kawanishi, 1999). In normal somatic cells, telomeres shorten with every cellular division. This makes telomere length measurement a robust marker of aging in many species, including humans and rodents, and this has been shown to be associated with longevity (Haussman et al., 2003; Heidinger et al., 2012). It is known that suboptimal *in utero* nutrition can lead to accelerated aging in a number of tissues (Tarry-Adkins and Ozanne, 2014). The high metabolic activity of skeletal muscle renders it particularly susceptible to oxidative stress; however, accelerated aging in skeletal muscle as a consequence of developmental programming has never been explored.

This study therefore aimed to investigate the effects of a poor maternal diet followed by accelerated postnatal growth upon skeletal muscle (vastus lateralis) of aging male rat offspring, focusing specifically upon telomere length, and indices of oxidative stress, antioxidant defense capacity and inflammation.

## RESULTS

In all cases, the reported data are expressed as mean±s.e.m.

### Anthropometrical data

Recuperated offspring were significantly (*P*<0.001; 6.3±0.3 g) smaller compared to controls (7.4±0.2 g) on day 3, and remained significantly (*P*<0.001) smaller at day 7 (13.4±0.6 vs 16.8±0.8 g). By 14 days of age, the recuperated offspring had undergone rapid postnatal catch-up growth and so were similar in weight to control offspring (33.7±0.7 g vs 34±1.7 g), and this was maintained at weaning (52.2±0.9 g vs 50.7±1.2 g) and at 12 months of age (920±29 g vs 956±25 g). These values reflect average male pup weight in the litter.

### Poor maternal nutrition and accelerated postnatal growth accelerated telomere shortening and increased DNA damage

**Fig. 1.**
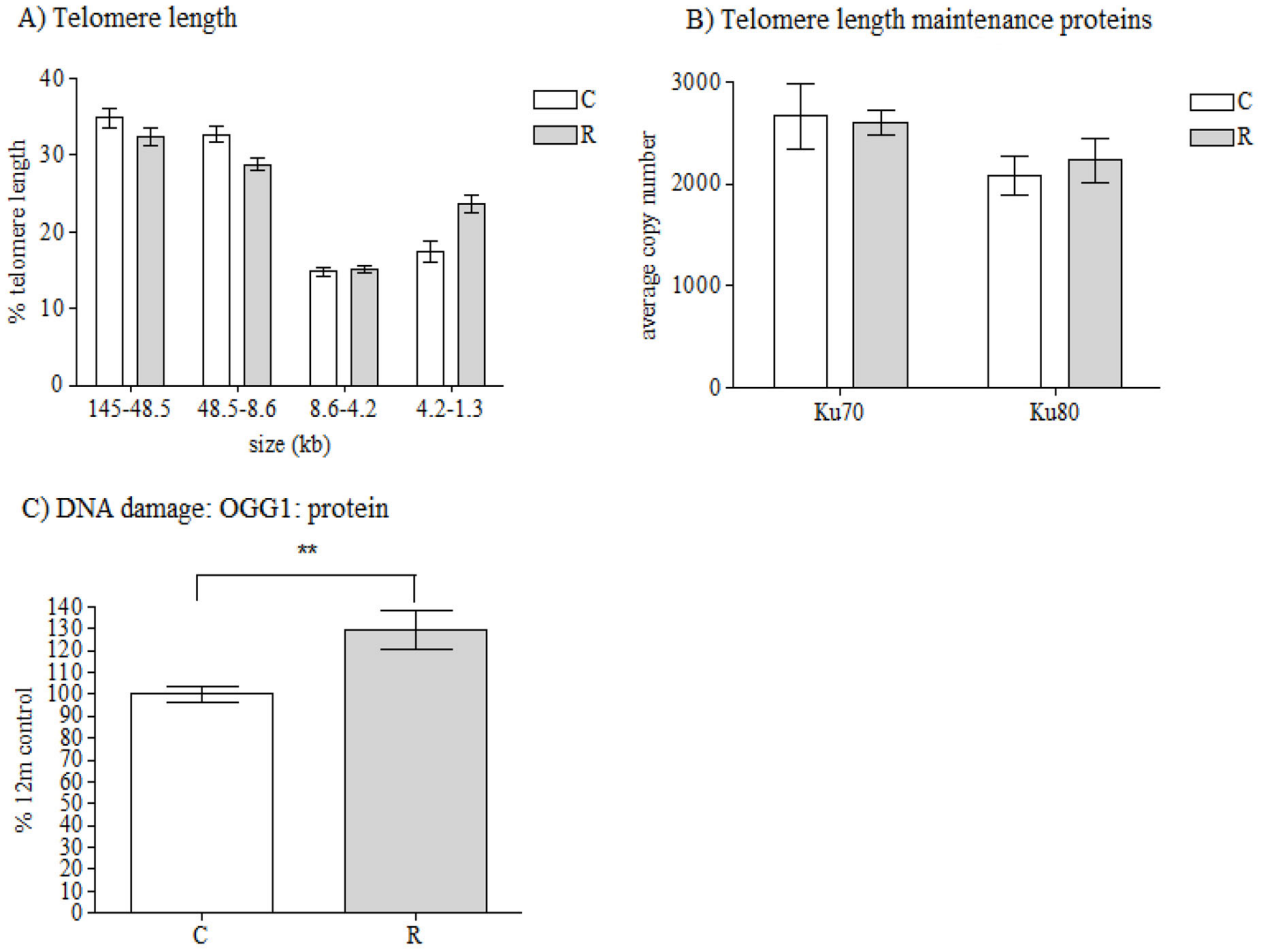
**Telomere length and markers of DNA damage.** The effect of *in utero* protein restriction and accelerated postnatal growth upon (A) telomere length (the percentage of telomeres at each length is shown), (B) mRNA expression of telomere-length maintenance proteins (*Ku70* and *Ku80*) and (C) protein expression of DNA-damage-repair protein (OGG1) in vastus lateralis skeletal muscle of 12-month-old male rats (shown as a percentage of the total amount in control rats). Results are expressed as mean±s.e.m. **P<0.01 (control versus recuperated). Statistics were calculated using a Student's *t*-test (two-tailed) and a linear regression model was used to analyze the telomere length data, which included effects of maternal diet (*P*<0.05), category of telomere length (*P*<0.001). C, control; R, recuperated. *n*=6 per group for telomere-length analysis and protein expression; *n*=8 per group for mRNA expression.

Vastus lateralis muscle from recuperated animals had fewer long telomeres and more short telomeres compared to control animals, with significant effects of both maternal diet (*P*<0.05) and telomere length category (*P*<0.01) (Fig. 1A). There was no significant effect of maternal diet upon mRNA expression of the telomere-length maintenance proteins *Ku70* and *Ku80* (Fig. 1B). However, increased (*P*<0.01) protein expression of the base excision-repair protein 8-oxoguanine-glycosylase (OGG1) was observed in recuperated animals compared to controls (Fig. 1C). There was no effect of maternal diet upon the mRNA levels of markers of cellular senescence, p53 (776±119 vs 789±99) or p21 (531±88 vs 370±32) (average copy number, control versus recuperated).

### Poor maternal nutrition and accelerated postnatal growth lead to increased skeletal-muscle oxidative stress

#### (a) NF-κB:

**Fig. 2.**
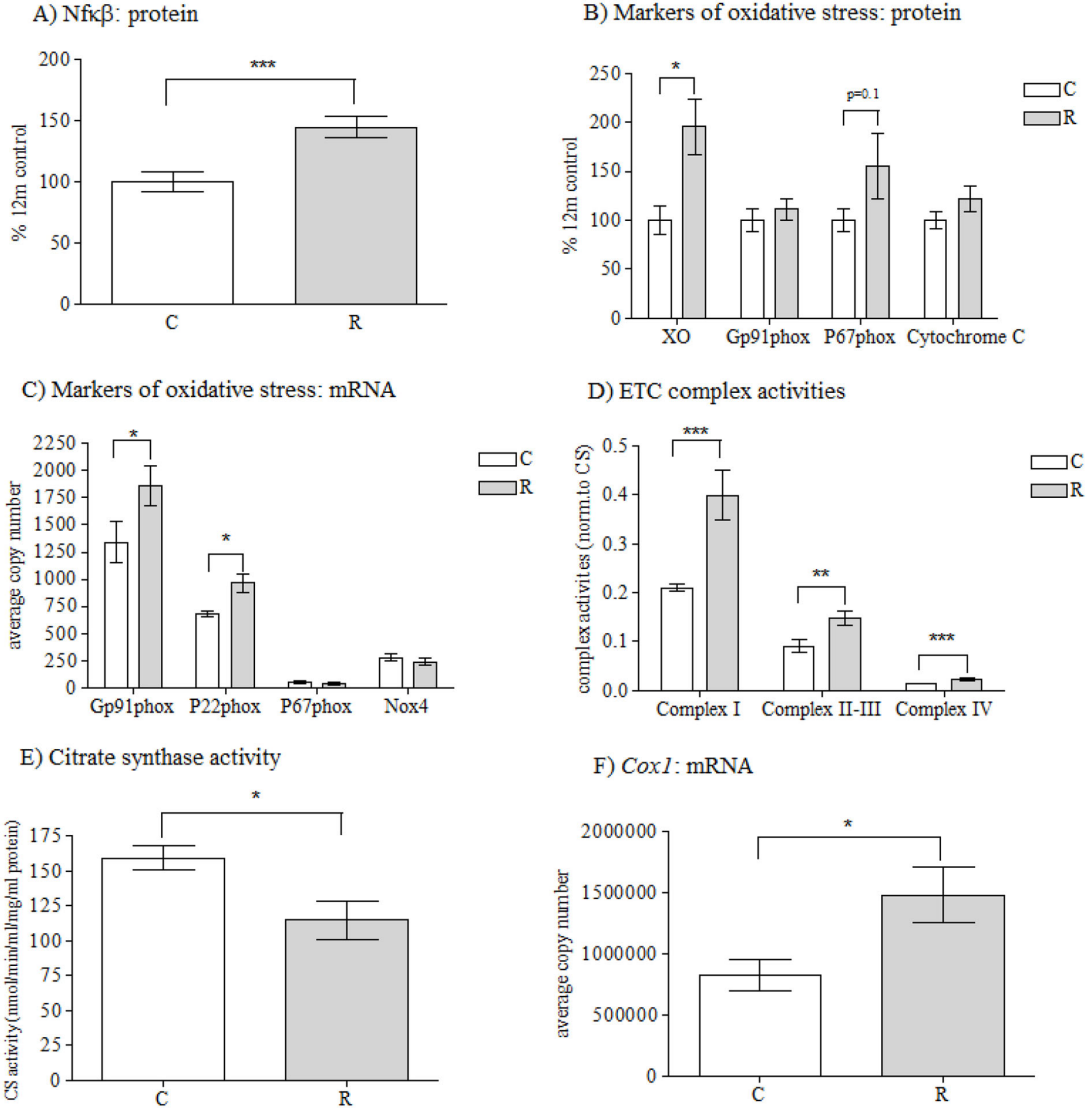
**Oxidative stress markers.** The effect of *in utero* protein restriction and accelerated postnatal growth upon protein expression of (A) NF-κB, (B) markers of oxidative stress (XO, Gp91^phox^, P67^phox^ and cytochrome *c*), (C) mRNA expression of *Gp91^phox^*, *P22^phox^*, *P67^phox^* and *Xo*, (D) ETC complex activity, (E) citrate synthase (CS) activity and (F) *Cox1* mRNA expression in vastus lateralis skeletal muscle in 12-month-old male rats. Results in A and B are shown as a percentage of the total amounts in control rats. Results are expressed as mean±s.e.m. *q<0.05 and **P<0.01, ***P<0.001 (control versus recuperated). C, control; CS, citrate synthase; R, recuperated. *n*=6 per group for protein expression; *n*=8 per group for mRNA expression analysis; *n*=10 per group for ETC complex activity analysis.

There was a significant effect of *in utero* maternal diet upon protein expression of NF-κB, with increased (*P*<0.001) NF-κB protein levels in recuperated offspring compared to controls (Fig. 2A). There was, however, no effect of maternal diet (control 442±60 vs recuperated 377±60 copy number) upon *Nf-κB1* gene expression. This suggests that the mechanism underlying the NF-κB dysregulated expression involves post-transcriptional regulation.

#### (b) NADPH oxidase 2 (NOX2), xanthine oxidase (XO) and cytochrome *c*:

XO protein expression was significantly (*P*<0.05) increased in recuperated offspring compared to controls and there was a trend towards an increase in P67^phox^ (*P*=0.1) (Fig. 2B); however cytochrome *c* protein expression was similar between groups (Fig. 2B). mRNA levels of the NOX2 protein-complex components *Gp91^phox^* (*P*<0.05) and *P22^phox^* (*P*<0.05) were significantly increased in recuperated animals compared to controls (Fig. 2C). Expression levels of *P67^phox^* and *Nox4* were unchanged between groups (Fig. 2C). Gene expression of *Xo* was also unchanged between groups (control 444±54, recuperated 344±40 copy number).

#### (c) Mitochondrial indices of ROS:

Levels of citrate synthase (CS), a marker of functional mitochondria, were decreased (*P*<0.05) in recuperated offspring compared to controls (Fig. 2D). Increased levels of complex I (*P*<0.001), linked complex II-III (*P*<0.01) and complex IV (*P*<0.001) activities were observed in recuperated offspring compared to controls (Fig. 2E). The increased complex II-III activity was not associated with any differences in coenzyme Q_9_ (CoQ_9_) (control 9675±660 vs recuperated 8410±695 pmol/mg protein). mRNA levels of Cytochrome *c* oxidase 1 (*Cox1*), a subunit of complex IV, was increased (*P*<0.05) in recuperated offspring compared to controls (Fig. 2F).

#### (d) Direct indices of Reactive Oxygen Species (ROS):

Markers of lipid peroxidation (4-Hydroxynonenal) and protein tyrosination (3-nitrotyrosine) were undetectable in skeletal muscle.

### Poor maternal nutrition and accelerated postnatal growth altered skeletal-muscle antioxidant defense capacity

**Fig. 3.**
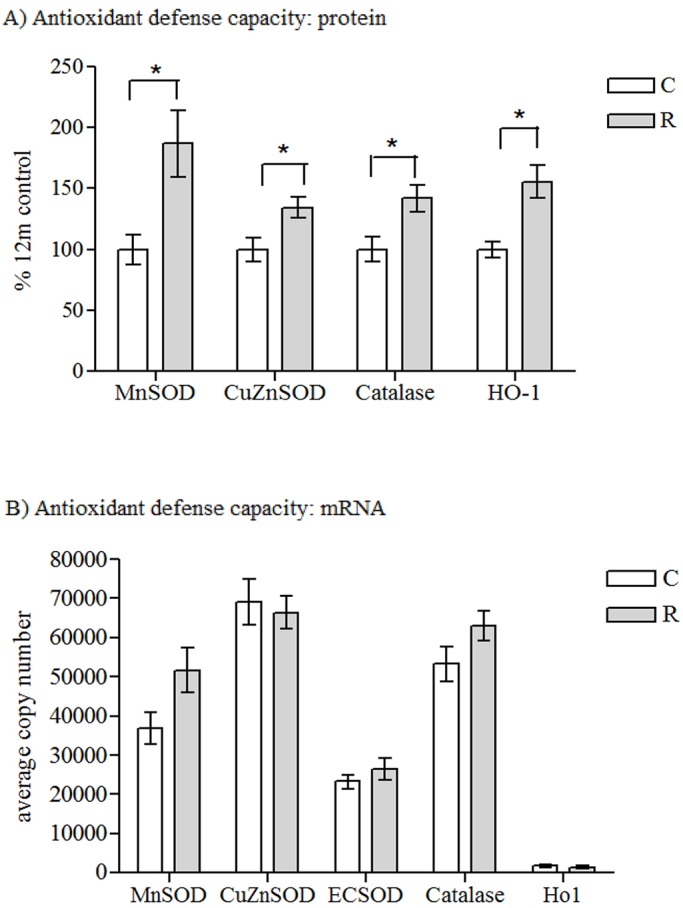
**Markers of antioxidant enzyme defense mechanisms.** The effect of *in utero* protein restriction and accelerated postnatal growth upon (A) protein expression (shown as a percentage of the total amounts in control rats) and (B) mRNA expression of antioxidant defense capacity in vastus lateralis skeletal muscle of 12-month-old male rats. Results are expressed as mean±s.e.m. *q<0.05 (control versus recuperated). Statistics were calculated using Student's *t*-test (two-tailed) and are reported after correction for multiple hypothesis testing where appropriate. C, control; R, recuperated. *n*=6 per group for protein analysis and *n*=8 per group for mRNA analysis.

Protein expression of the antioxidant enzymes manganese superoxide dismutase (MnSOD) (*P*<0.05), copper-zinc superoxide dismutase (CuZnSOD) (*P*<0.05), catalase (*P*<0.05) and heme oxygenase-1 (HO1) (*P*<0.05) were increased in recuperated offspring compared to controls (Fig. 3A). Protein expression of peroxiredoxin-1 (PRDX1), peroxiredoxin-3 (PRDX3) and glutathione reductase (GR) were unaffected by maternal diet (Table 1). mRNA expression of *MnSOD*, *CuZnSOD*, extracellular superoxide dismutase (*ECSOD*), catalase and *Hmox1* were unaffected by maternal diet (Fig. 3B).

**Table 1.**
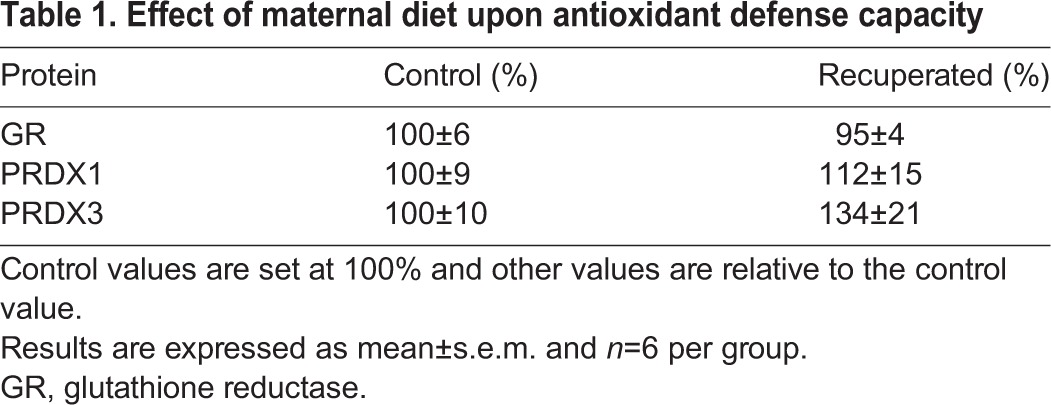
**Effect of maternal diet upon antioxidant defense capacity**

### NF-κB1 correlates with antioxidant defense and oxidant capacity

**Fig. 4.**
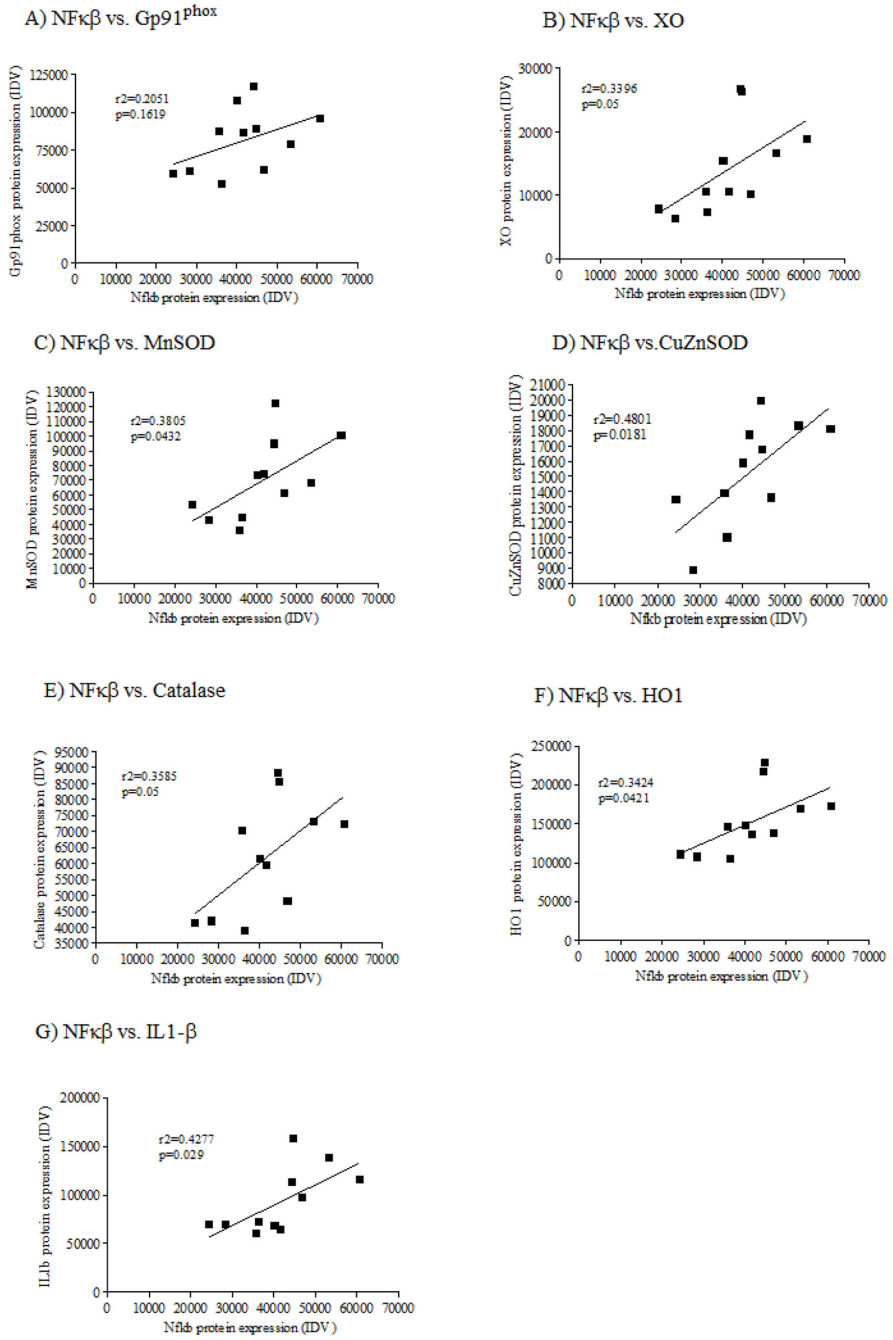
**Correlations of NF-κB protein expression with markers of oxidative stress, antioxidant enzymes and markers of inflammation.** The effect of *in utero* protein restriction and accelerated postnatal growth upon correlations of protein expression of NF-κB versus (A) Gp91^phox^, (B) XO, (C) MnSOD, (D) CuZnSOD, (E) catalase, (F) HO1 and (G) IL1β in vastus lateralis skeletal muscle of 12-month-old male rats. *P*-values are shown in the graphs (NF-κB versus antioxidants). Statistics were calculated using a Student's *t*-test (two-tailed). Results are expressed as mean±s.e.m. *n*=6 per group. IDV, integrated density value.

Positive correlations were observed between NF-κB1 protein expression and XO (*P*=0.05; *r*^2^=0.3596), MnSOD (*P*=0.0432; *r*^2^=0.3805), CuZnSOD (*P*=0.0181; *r*^2^=0.4801), catalase (*P*=0.05; *r*^2^=0.3585), HO1 (*P*=0.048; *r*^2^=0.3590) and IL1β (*P*=0.029; *r*^2^=0.4277). The correlation between Gp91^phox^ and NF-κB1 was non-significant (*P*=0.1619; *r*^2^=0.2051) (Fig. 4A-F).

### Poor maternal nutrition and accelerated postnatal growth lead to altered markers of inflammation

**Fig. 5.**
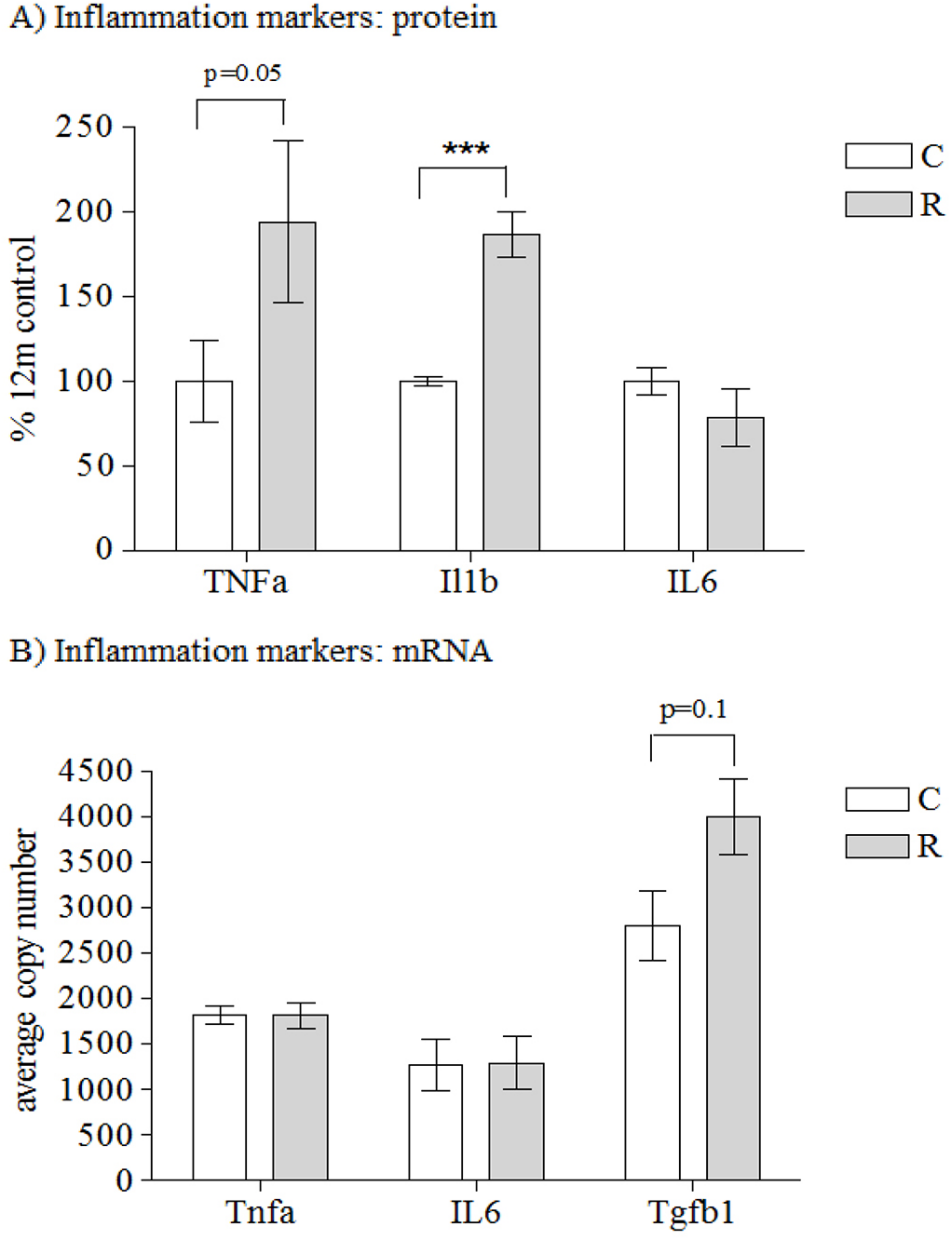
**Inflammatory markers.** The effect of *in utero* protein restriction and accelerated postnatal growth upon (A) protein expression of inflammation markers (TNFα, IL1β and IL6; shown as a percentage of the total amounts in control rats) and (B) mRNA expression of *Tnfα*, *Il6* and *Tgfβ1* in vastus lateralis skeletal muscle of 12-month-old male rats. Results are expressed as mean±s.e.m. ***q<0.001 (control versus recuperated). Statistics were calculated using Student's *t*-test (two-tailed) and are reported after correction for multiple hypothesis testing where appropriate. C, control; R, recuperated. *n*=6 per group for protein expression and *n*=8 per group for mRNA expression.

TNFα (*P*=0.05) and IL1β (*P*<0.001) protein levels were increased in recuperated offspring compared to controls; however, IL6 protein expression was similar between groups (Fig. 5A). No effect of maternal diet was observed upon *Tnfα* or *Il6* mRNA levels; however, there was a trend toward increased *Tgfβ1* mRNA expression levels (*P*=0.11) in recuperated offspring compared to controls (Fig. 5B).

## DISCUSSION

The aging process is associated with a decline in muscle ‘fitness’, with distinct muscle mass decline and loss of muscle strength occurring from 40 years of age in humans (Keller., 2013). This is becoming of increasing concern in an aging population. The environment to which an individual is exposed *in utero* and in early life has also been shown to have an effect on skeletal muscle in old age; skeletal muscle from elderly monozygotic twins, in which the lower-birth-weight twin developed type 2 diabetes (TD2) in later life, had perturbations in glycogen metabolism and insulin resistance, which are not apparent in young monozygotic twins (Poulsen et al., 2007). An accelerated postnatal growth trajectory in low-birth-weight children is also known to reduce physical fitness in pre-pubescent children (van Deutekom et al., 2015). We have shown previously using an animal model that low birth weight followed by accelerated postnatal growth leads to a reduction in lifespan (Hales et al., 1996). Low birth weight alone without postnatal catch-up growth did not impact on lifespan (Hales et al., 1996). It is unknown whether low birth weight and accelerated early postnatal growth impacts on skeletal muscle aging. We addressed this knowledge gap using a well-established rat model of maternal protein restriction, which generates low-birth-weight offspring and accelerated postnatal growth (recuperated), by cross-fostering to control-fed mothers.

Accelerated telomere shortening, a robust marker of cellular aging, was observed in aged skeletal muscle from recuperated rats compared to control animals, suggesting that low birth weight and rapid postnatal growth causes accelerated skeletal-muscle aging. This was not associated with premature cellular senescence: two markers of cellular senescence, p53 and p21, were unaltered at the mRNA level; however, changes at the protein level cannot be disregarded. Ku70 and Ku80, two of the major telomere-length maintenance proteins, which are also instrumental in non-homologous end-joining (NHEJ) DNA repair (a mechanism that repairs double-stranded DNA breaks), were unchanged at the mRNA level. However, we cannot discount the possibility that changes may occur at the protein level: there is a lack of antibodies to these molecules that work well in muscle. OGG1 the major enzyme involved in the excision of 8-oxo-7,8-dihydroguanine (8-oxodG) DNA base lesions via the base excision repair (BER) mechanism, which is key in repairing oxidative base damage specifically in telomeres, was increased at the protein level in recuperated offspring. It is worthwhile to note that we previously reported an increase in OGG1 protein expression in hearts from recuperated rats (Tarry-Adkins et al., 2012).

The increase in skeletal-muscle OGG1 is particularly noteworthy given that recuperated offspring demonstrated a strong oxidative stress phenotype with increased XO protein expression, elevated components of the NADPH oxidase-2 (Gp91^phox^, P22^phox^ and P67^phox^) protein complex at both the protein and mRNA level and increased NF-κB protein expression. This suggests that the observed upregulation of OGG1 is a compensatory mechanism to attempt to repair the oxidative base damage.

Skeletal muscle is one of the most aerobically and metabolically active tissues in the body, and is therefore extremely mitochondrially rich, and a major source of oxidative stress. Therefore, we investigated muscle mitochondria as a potential source of ROS in vastus lateralis muscle of the recuperated offspring. Evidence of mitochondrial dysfunction was observed in the muscle of recuperated offspring by a reduction in CS activity (a marker for functional intact mitochondria), and increased complex I, linked complex II-III and complex IV electron transport chain (ETC) activity. It has been shown that, in states of ‘mitochondrial hyperactivity’ (in which ETC activities are upregulated), the ETC generates pathologically high ΔΨ_m_ levels, and this mitochondrial hyperpolarization leads to an exponential increase in ROS generation at membrane potentials exceeding 140 mV. This has been demonstrated in many pathologies, including lupus erythematosus (Doherty et al., 2014), rubella infection (Claus et al., 2013) and in multiple sclerosis lesions (Mahad et al., 2009). mRNA levels of *Cox1*, one of the three mitochondrially encoded subunits of complex IV of the ETC (Heilbronn et al., 2007), was also upregulated in the skeletal muscle of aged recuperated offspring. Taken together, evidence of a reduced number of functional mitochondria and increased ETC activity suggests that the recuperated muscle mitochondria might have to compensate for fewer mitochondria by increasing the activity of ETC complexes to generate sufficient ATP, which in turn produces more ROS. This ‘vicious cycle’ of events is hypothesized to occur in the normal aging process (Wang et al., 2013), which is accelerated further in skeletal muscle of recuperated offspring. As a point of interest, vastus lateralis muscle is reported to be either white or a mixture of red and white fibers (both fast and slow twitch) and therefore has differences in mitochondrial density compared to other muscle types, such as the soleus muscle which is predominantly made from red (slow twitch) muscle fibers; therefore, investigations into different muscle types with regards to their mitochondrial content and fuel partitioning profiles would be of great interest for future study.

Recuperated offspring also showed consistently upregulated antioxidant defense capacity, particularly at the protein level; however, *MnSOD* was also increased at the mRNA level in recuperated offspring. Given the increased oxidative stress, it is highly likely that this increase is a compensatory mechanism to deal with the increased oxidative damage. We observed a similar compensatory phenotype of increased antioxidant capacity in hearts from recuperated rat offspring (Tarry-Adkins et al., 2012).

NF-κB is a transcription factor whose activation causes severe muscle atrophy in mice (Cai et al., 2004). It is a master regulator of ROS and is known to drive the upregulation of many pro-oxidants, including Gp91^phox^ (which also might be involved in a positive-feedback loop in which NF-κB activation by oxidative stress leads to further radical production via NADPH oxidase) (Anrather et al., 2006) and XO (Xu et al., 1996). One of the most important ways in which NF-κB activity influences ROS levels is via increased expression of antioxidant enzymes (Morgan and Lui, 2011). MnSOD is the most well-known NF-κB target (Kairisalo et al., 2007); however, CuZnSOD has also been implicated as being an NF-κB target (Rojo et al., 2004) and *HO1* is also upregulated by NF-κB in situations of increased ROS (Lavrovsky et al., 1994). Consistent with NF-κB being a driver of the increase in antioxidant defense capacity, we demonstrated significant positive correlations between NF-κB versus XO, MnSOD, CuZnSOD and HO1 protein expression in this study.

The skeletal muscle from recuperated offspring also demonstrated a pro-inflammatory phenotype, with increased TNFα and IL1β protein expression and increased *Tgfβ1* mRNA expression. NF-κB is also a master regulator of inflammation and, interestingly, TNFα and IL1β can regulate MnSOD and can also cause rapid activation and nuclear translocation of NF-κB (Beg et al., 1993; Jones et al., 1997).

In conclusion, we have shown evidence for accelerated aging as a consequence of suboptimal nutrition and provide a molecular basis through which this can occur. This includes accelerated telomere shortening and increased DNA damage, which was associated with a strong oxidative stress phenotype, a compensatory increased antioxidant defense capacity and inflammation – all of which may be regulated by NF-κB signaling. These findings provide an explanation of why some individuals are at greater risk of developing age-associated muscular dysfunction than others. Given that oxidative stress is a major phenotype in this model, this study provides a strong rationale for a targeted postnatal antioxidant intervention as a potentially safe and cost-effective therapy in at-risk individuals.

## MATERIALS AND METHODS

### Animal experimentation

All procedures involving animals were conducted under the British Animals (Scientific Procedures) Act (1986) and underwent ethical review by the University of Cambridge Animal Welfare and Ethical Review Board. Stock animals were purchased from Charles River. Dams were produced from in-house breeding from stock animals, and each was paired with a different stock male for mating. Pregnant Wistar rats (*rattus norvegicus*) were maintained at room temperature in specific pathogen-free (SPF) housing using individually ventilated cages with environmental enrichment. The dams were maintained on a 20% protein diet (control) or, an isocaloric low protein (LP) (8%) diet, as previously described (Snoeck et al., 1990). Access to diets and water was provided *ad libitum*. All animals used in this study were SPF-housed individually at 22°C on a controlled 12:12-h light-dark cycle. Diets were purchased from Arie Blok (Woerden, The Netherlands).

The day of birth was recorded as day 1 of postnatal life. Pups born to LP diet-fed dams were cross-fostered to control-fed mothers on postnatal day 3, in order to create a recuperated litter. Each recuperated litter was standardized to four male pups at random to maximize their plane of nutrition. The control group was the offspring of mothers fed the 20% protein diet and suckled by 20% protein-fed dams. Each control litter was culled to eight pups as a standard. Animals in this group were suckled by their own dams. To minimize stress to the animals when cross-fostered, pups were transferred with some of their own bedding. Body weights were recorded at postnatal days 3, 7, 14 and 21, and at 12 months. For time points up until 21 days of age, these reflect average male pup weight in the litter. At 21 days, two males per litter were weaned in their home-cage onto standard laboratory chow fed *ad libitum* (Special Diet Services) and were maintained on this diet until 12 months of age. All animals were killed by CO_2_ asphyxiation at approximately 10 am. At post-mortem, vastus lateralis tissue was removed, weighed and snap-frozen in liquid nitrogen and then stored at −80°C until analysis. Ten litters per group were used in this study; this was based on power calculations. In all cases, *n* refers to the number of litters (with one animal used from each litter).

### Reagents

All general reagents for western blotting were purchased from Sigma (Poole, UK), except for the antibodies, which are detailed in the Protein analysis section. All general reagents for gene expression analysis were purchased from Applied Biosystems (Warrington, UK) and all general reagents for CoQ_9_ and ETC activities were sourced from Sigma (Poole, UK).

### Protein analysis

Protein was extracted from samples of vastus lateralis muscle tissue and assayed as described previously (Tarry-Adkins et al., 2016). Protein (20 µg) was loaded onto 10%, 12% or 15% polyacrylamide gels, dependent upon the molecular weight of the protein to be measured. The samples were electrophoresed and transferred to polyvinylidene fluoride membranes (Tarry-Adkins et al., 2016), and detected using the following dilutions of primary antibody: OGG1 (Novus Biologicals, Abingdon, UK; cat. no.: NB100-106, 1:500), XO (Santa-Cruz, Wimbledon, Middlesex, UK; cat. no.: SC-20991, 1:200), Gp91^phox^ (ProteinTech, Cambridge, UK; cat. no.: 19013-1-AP, 1:1000), P67^phox^ (ProteinTech, Cambridge, UK; cat. no.: 15551-1-AP, 1:1000), cytochrome *c* (Abcam, Cambridge, UK; cat. no.: Ab90529, 1:2000), MnSOD (Upstate, Watford, UK; cat. no.: 06-984, lot 26654, 1:1000), CuZnSOD (ProteinTech, Cambridge, UK; cat. no.: 10269-1-AP, 1:1000), TNFα (Cell Signaling Technology, Danvers, MA, USA; cat. no.: 11948S). NF-κB (cat. no.: Ab89060), catalase (cat. no.: Ab1877-10), GR (cat. no.: Ab16801), PRDX1 (cat. no.: Ab15571), PRDX3 (cat. no.: 6751), IL6 (cat. no.: Ab6672), IL1β (cat. no.: Ab9722) and HO1 (cat. no.: Ab6672) were all diluted 1:1000 and purchased from Abcam (Cambridge, UK). All antibodies used anti-rabbit IgG secondary antibodies from Cell Signaling Technology (Danvers, MA, USA) at a dilution of 1:2000. Equal protein loading was confirmed by staining electrophoresed gels with Coomassie blue (Bio-Rad, Hemel Hempstead, UK) to visualize total protein.

### Gene expression

RNA was extracted using an RNeasy Plus mini kit (Qiagen, Manchester, UK) following the manufacturer's instructions. A DNase digestion step was performed in order to ensure no genomic DNA contamination. RNA (1 µg) was used to synthesize cDNA using oligo-dT primers and M-MLV reverse transcriptase (Promega, Southampton, UK). Gene expression was determined using custom-designed primers (Sigma, Poole, UK) and SYBR Green reagents (Applied Biosystems, Warrington, UK). Primer sequences are presented in Table 2. Quantification of gene expression was performed using a Step One Plus RT-PCR machine (Applied Biosystems, Warrington, UK). Equal efficiency of the reverse transcription of RNA from all groups was confirmed through quantification of expression of the housekeeping gene *Ppia.* Expression of *Ppia* did not differ between groups (effect of maternal diet *P*=0.99; control 37±6, recuperated 39±6 average copy number). Sample sizes were *n*=8 per group.

**Table 2.**
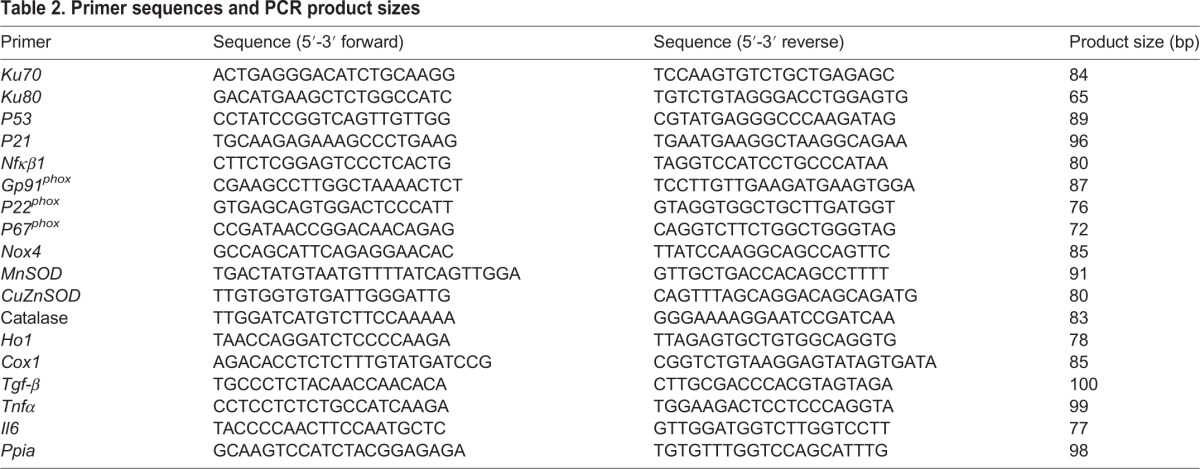
**Primer sequences and PCR product sizes**

### Mitochondrial ETC complex activities and CoQ measurement

Activities of complex I (NADH: ubiquinone reductase; EC 1.6.5.3), complex II-III (succinate: cytochrome *c* reductase; EC 1.3.5.1+EC 1.10.2.2) and complex IV (cytochrome oxidase; EC 1.9.3.1) as well as citrate synthase (CS; EC 1.1.1.27) were assayed as described previously (Tarry-Adkins et al., 2016). Vastus lateralis CoQ_9_ was quantified by reverse phase high-performance liquid chromatography (HPLC) with UV detection at 275 nm as described previously (Tarry-Adkins et al., 2016).

### 4-hydroxynonenal (4-HNE) and 3-nitrotyrosine (3-NT) analysis

Protein nitrotyrosination was assayed using a 3-Nitrotyrosine ELISA kit (MitoSciences, Cambridge, UK), according to the manufacturer's instructions. 4-HNE (a marker of lipid peroxidation) was analyzed using an OxiSelect HNE Adduct ELISA kit (Cambridge Biosciences), according to the manufacturer's instructions.

### Statistical analysis

Maternal-diet effects were compared between groups using Student's *t*-test for single hypotheses. In order to correct for multiple hypothesis testing where relevant, *P*-values were transformed to take account of the false discovery rates using the p.adjust function in R stats package. A linear regression model was used to analyze the telomere length data, which included effects of maternal diet, category of telomere length and an interaction term between these. Data are represented as mean±s.e.m. All statistical analyses were performed using either Statistica 7 software (Statsoft Inc., Bracknell, UK) or R version 3.1.0 (R Foundation for Statistical Computing, Vienna, Austria). Where *P*-values or adjusted *P*-values are reported, an alpha level <0.05 was considered statistically significant. Data was checked for normal distribution. In all cases, *n* refers to the number of litters (with one animal used from each litter).

## References

[DMM026591C2] Anrather J., Racchumi G. and Iadecola C. (2006). NF-kappa beta regulates phagocytic NADPH oxidase by inducing the expression of gp91phox. *J. Biol. Chem.* 281, 5657-5667. 10.1074/jbc.M506172200 16407283

[DMM026591C3] Barker D. J. P., Osmond C., Winter P. D., Margetts B. and Simmonds S. J. (1989). Weight in infancy and death from ischaemic heart disease. *Lancet* 334, 577-580. 10.1016/S0140-6736(89)90710-1 2570282

[DMM026591C4] Barker D. J. P., Hales C. N., Fall C. H. D., Osmond C., Phipps K. and Clark P. M. S. (1993). Type 2 (non-insulin-dependent) diabetes mellitus, hypertension and hyperlipidaemia (syndrome X): relation to reduced fetal growth. *Diabetologia* 36, 62-67. 10.1007/BF00399095 8436255

[DMM026591C5] Beauchamp B., Ghosh S., Dysart M. W., Kanaan G. N., Chu A., Blais A., Rajamanickam K., Tsai E. C., Patti M. E. and Harper M. E. (2015). Low birth weight is associated with adiposity, impaired skeletal muscle energetics and weight loss resistance in mice. *Int. J. Obes.* 39, 702-711. 10.1038/ijo.2014.120 PMC4326251 25091727

[DMM026591C6] Beg A. A., Finco T. S., Nantermet P. V. and Baldwin A. S. (1993). Tumor necrosis factor and interleukin-1 lead to phosphorylation and loss of IkBa: a mechanism for NF-kB activation. *Mol. Cell. Biol.* 13, 3301-3310. 10.1128/MCB.13.6.3301 8497253 PMC359784

[DMM026591C7] Cai D., Frantz J. D., Tawa N. E., Mendelez P. A., Oh B.-C., Lidov H. G. W., Hasselgren P.-O., Frontera W. R., Lee J., Glass D. J. et al. (2004). IKKκ/NF-κβ activation causes severe muscle wasting in mice. *Cell* 119, 285-298. 10.1016/j.cell.2004.09.027 15479644

[DMM026591C8] Claus C., Schonefield K., Hubner D., Chey S., Reibetanz U. and Liebert U. G. (2013). Activity increase in respiratory chain complexes by rubella virus with marginal induction of oxidative stress. *J. Virol.* 87, 8481-8492. 10.1128/JVI.00533-13 23720730 PMC3719815

[DMM026591C9] Confortim H. D., Jeronimo L. C., Centanaro L. A., Pinheiro P. F., Matheus S. M. and Torrejais M. M. (2015). Effects of age and maternal protein restriction on the muscle fibers morphology and neuromuscular junctions of rats after nutritional recovery. *Micron* 71, 7-13. 10.1016/j.micron.2014.12.006 25597842

[DMM026591C10] Confortim H. D., Jeronimo L. C., Centanaro L. A., Pinheiro P. F., Matheus S. M. and Torrejais M. M. (2016). Maternal protein restriction during pregnancy and lactation affects the development of muscle fibers and neuromuscular junctions in rats. *Muscle Nerve*10.1002/mus.25187 [Epub ahead of print] doi:10.1002/mus.25187 10.1002/mus.25187 27171684

[DMM026591C11] Cruz-Jentoft A. J., Baeyens J. P., Bauer J. M., Boirie Y., Cederholm T., Landi F., Martin F. C., Michel J.-P., Rolland Y., Schneider S. M. et al. (2010). Sarcopenia: European consensus on definition and diagnosis: Report of the European Working Group on Sarcopenia in Older People. *Age Ageing* 39, 412-423. 10.1093/ageing/afq034 20392703 PMC2886201

[DMM026591C12] Desai M., Crowther N. J., Lucas A. and Hales C. N. (1996). Organ-selective growth in the offspring of protein-restricted mothers. *Br. J. Nutr.* 76, 591-603. 10.1079/bjn19960065 8942365

[DMM026591C13] Doherty E., Oaks Z. and Perl A. (2014). Increased mitochondrial electron transport chain activity at complex I is regulated by *N*-acetylcysteine in Lymphocytes of patients with systemic lupus erythematosus. *Antioxid. Redox. Signal.* 21, 56-65. 10.1089/ars.2013.5702 24673154 PMC4048573

[DMM026591C14] Hales C. N. and Barker D. J. P. (1992). Type 2 (non-insulin-dependent) diabetes mellitus: the thrifty phenotype hypothesis. *Diabetologia* 35, 595-601. 10.1007/BF00400248 1644236

[DMM026591C47] Hales C. N., Desai M., Ozanne S. E. and Hales C. N. (1996). Fishing in the stream of diabetes: from measuring insulin to the control of fetal organogenesis. *Biochem. Soc. Trans.* 24, 341-350. 10.1042/bst0240341 8736760

[DMM026591C15] Haussman M. F., Winkler D. W., O'Reilly K. M., Huntington C. E., Nisbet I. C. T. and Vleck C. M. (2003). Telomeres shorten more slowly in long-lived birds and mammals than in short-lived ones. *Proc. R. Soc. B Biol. Sci.* 270, 1387-1392. 10.1098/rspb.2003.2385 PMC1691385 12965030

[DMM026591C16] Heidinger B. J., Blount D. J., Boner W., Griffiths K., Metcalfe N. B. and Monaghan P. (2012). Telomere length in early life predicts lifespan. *Proc. Natl. Acad. Sci. USA* 109, 1743-1748. 10.1073/pnas.1113306109 22232671 PMC3277142

[DMM026591C17] Heilbronn L. K., Gan S. K., Turner N., Campbell L. V. and Chisholm D. J. (2007). Markers of mitochondrial biogenesis and metabolism are lower in overweight and obese insulin-resistant subjects. *J. Clin. Endocrinol. Metab.* 92, 1467-1473. 10.1210/jc.2006-2210 17244782

[DMM026591C19] Jansson N., Pettersson J., Haafiz A., Ericsson A., Palmberg I., Tranberg M., Ganapathy V., Powell T. L. and Jansson T. (2006). Down-regulation of placental transport of amino acids precedes the development of intrauterine growth restriction in rats fed a low protein diet. *J. Physiol.* 576, 935-946. 16916910 10.1113/jphysiol.2006.116509 PMC1892642

[DMM026591C20] Jones P. L., Ping D. and Boss J. M. (1997). Tumor necrosis factor alpha and interleukin-1 beta regulate the murine manganese superoxide dismutase gene through a complex intronic enhancer involving C/EBP-beta and NF-kappaB. *Mol. Cell. Biol.* 17, 6970-6981. 10.1128/MCB.17.12.6970 9372929 PMC232554

[DMM026591C21] Kairisalo M., Korhonen L., Blomgren K. and Lindholm D. (2007). X-linked inhibitor of apoptosis protein increases mitochondrial antioxidants through NF-kappaB activation. *Biochem. Biophys. Res. Commun.* 364, 138-144. 10.1016/j.bbrc.2007.09.115 17936246

[DMM026591C22] Keller K. (2013). Strength and muscle mass loss with aging process. Age and strength loss. *Muscles Tendons Ligaments J.* 24, 346-350. PMC3940510 24596700

[DMM026591C23] Lavrovsky Y., Schwartzman M. L., Levere R. D., Kappas A. and Abraham N. G. (1994). Identification of binding sites for transcription factors NFkappa B and AP-2 in the promoter region of the human heme oxygenase 1 gene. *Proc. Natl. Acad. Sci. USA* 91, 5987-5991. 10.1073/pnas.91.13.5987 8016102 PMC44122

[DMM026591C24] Mahad D. J., Ziabreva I., Campbell G., Lax N., White K., Hanson P. S., Lassman H. and Turnbull D. M. (2009). Mitochondrial changes within axons in multiple sclerosis. *Brain* 132, 1161-1174. 10.1093/brain/awp046 19293237 PMC3605917

[DMM026591C25] Marzetti E., Calvani R., Cesari M., Buford T. W., Lorenzi M., Behnke B. J. and Leeuwenburgh C. (2013). Mitochondrial dysfunction and sarcopenia of aging: from signaling pathways to clinical trials. *Int. J. Biochem. Cell Biol.* 45, 2288-2301. 10.1016/j.biocel.2013.06.024 23845738 PMC3759621

[DMM026591C26] Morgan M. J. and Lui Z.-G. (2011). Crosstalk of reactive oxygen species and NF-κβ signaling. *Cell Res.* 21, 103-115. 10.1038/cr.2010.178 21187859 PMC3193400

[DMM026591C27] Mortensen O. H., Olsen H. L., Frandsen L., Nielsen P. E., Nielsen F. C., Grunnet N. and Quistorff B. (2010). A maternal low protein diet has pronounced effects on mitochondrial gene expression in offspring liver and skeletal muscle; protective effects of taurine. *J. Biomed. Sci.* 17 Suppl. 1, S38 10.1186/1423-0127-17-s1-s38 20804614 PMC2994375

[DMM026591C28] Oikawa S. and Kawanishi S. (1999). Site-specific DNA damage at GGG sequence by oxidative stress may accelerate telomere shortening. *FEBS Lett.* 453, 365-368. 10.1016/S0014-5793(99)00748-6 10405177

[DMM026591C30] Pantham P., Rosario F. J., Nijland M., Cheung A., Nathanielsz P. W., Powell T. L., Galan H. L., Li C. and Jansson T. (2015). Reduced placental amino acid transport in response to maternal nutrient restriction in the baboon. *Am. J. Physiol. Regul. Integr. Comp. Physiol.* 309, R740-R746. 10.1152/ajpregu.00161.2015 26246504 PMC4666932

[DMM026591C31] Patel H. P., Jameson K. A., Syddall H. E., Martin H. J., Stewart C. E., Cooper C. and Sayer A. A. (2012). Developmental influences, muscle morphology, and sarcopenia in community-dwelling older men. *J. Gerontol. A. Biol. Sci. Med. Sci.* 67A, 82-87. 10.1093/gerona/glr020 21357193

[DMM026591C32] Poulsen P., Wojtaszewski J. F. P., Richter E. A., Beck-Nielsen H. and Vaag A. (2007). Low birth weight and zygosity status is associated with defective muscle glycogen and glycogen synthase regulation in elderly twins. *Diabetes* 56, 2710-2714. 10.2337/db07-0155 17698598

[DMM026591C33] Ray P. D., Huang B.-W. and Tsuji Y. (2012). Reactive oxygen species (ROS) homeostasis and redox regulation in cellular signaling. *Cell Signal.* 24, 981-990. 10.1016/j.cellsig.2012.01.008 22286106 PMC3454471

[DMM026591C34] Rojo A. I., Salinas M., Martin D., Perona R. and Cuadrado A. (2004). Regulation of Cu/Zn-superoxide dismutase expression via the phosphatidylinositol 3 kinase/Akt pathway and nuclear factor-kappaB. *J. Neurosci.* 24, 7324-7334. 10.1523/JNEUROSCI.2111-04.2004 15317858 PMC6729771

[DMM026591C35] Sayer A. A., Stewart C., Patel H. and Cooper C. (2010). The developmental origins of sarcopenia: from epidemiological evidence to underlying mechanisms. *J. Develop. Orig. Health Dis.* 1, 150-157. 10.1017/S2040174410000097 25141783

[DMM026591C36] Snoeck A., Remacle C., Reusens B. and Hoet J. J (1990). Effect of a low protein diet during pregnancy on the fetal rat endocrine pancreas. *Biol. Neonate* 57, 107-118. 10.1159/000243170 2178691

[DMM026591C37] Tarry-Adkins J. L. and Ozanne S. E. (2014). The impact of early nutrition on the ageing trajectory. *Proc. Nutr. Soc.* 73, 289-301. 10.1017/S002966511300387X 24411102

[DMM026591C38] Tarry-Adkins J. L., Martin-Gronert M. S., Fernandez-Twinn D. S., Hargreaves I., Alfaradhi M. Z., Land J. M., Aiken C. E. and Ozanne S. E. (2012). Poor maternal nutrition followed by accelerated postnatal growth leads to alterations in DNA damage and repair, oxidative and nitrosative stress, and oxidative defense capacity in rat heart. *FASEB J.* 27, 379-390. 10.1096/fj.12-218685 23024373

[DMM026591C39] Tarry-Adkins J. L., Fernandez-Twinn D. S., Hargreaves I. P., Neergheen V., Aiken C. E., Martin-Gronert M. S., McConnell J. M. and Ozanne S. E. (2016). Coenzyme Q10 prevents hepatic fibrosis, inflammation, and oxidative stress in a male rat model of poor maternal nutrition and accelerated postnatal growth. *Am. J. Clin. Nutr.* 103, 579-588. 10.3945/ajcn.115.119834 26718412 PMC4733260

[DMM026591C40] Valko M., Leibfritz D., Moncol J., Cronin M. T. D., Mazur M. and Telser J. (2007). Free radicals and antioxidants in normal physiological functions and human disease. *Int. J. Biochem. Cell Biol.* 39, 44-84. 10.1016/j.biocel.2006.07.001 16978905

[DMM026591C41] van Deutekom A. W., Chinapaw M. J. M., Vrijkotte T. G. M. and Gemke R. J. B. J. (2015). The association of birth weight and infant growth with physical fitness at 8-9 years of age- the ABCD study. *Int. J. Obes.* 39, 593-600. 10.1038/ijo.2014.204 25468828

[DMM026591C42] Wang C.-H., Wu S.-B., Wu Y.-T. and Wei Y.-H. (2013). Oxidative stress response elicited by mitochondrial dysfunction: implication in the pathophysiology of aging. *Exp. Biol. Med.* 238, 450-460. 10.1177/1535370213493069 23856898

[DMM026591C43] Xu P., Huecksteadt T. P. and Hoidal J. R. (1996). Molecular cloning and characterization of the human xanthine dehydrogenase gene (XDH). *Genomics* 34, 173-180. 10.1006/geno.1996.0262 8661045

[DMM026591C44] Zambrano E., Ibáñez C., Martínez-Samayoa P. M., Lomas-Soria C., Durand-Carbajal M. and Rodríguez-González G. L. (2016). Maternal obesity: lifelong metabolic outcomes for offspring from poor developmental trajectories during the perinatal period. *Arch. Med. Res.* 47, 1-12. 10.1016/j.arcmed.2016.01.004 26827819

[DMM026591C45] Zhu M.-J., Ford S. P., Nathanielsz P. W. and Du M. (2004). Effect of maternal nutrient restriction in sheep on the development of fetal skeletal muscle. *Biol. Reprod.* 71, 1968-1973. 10.1095/biolreprod.104.034561 15317692

[DMM026591C46] Zhu M. J., Ford S. P., Means W. J., Hess B. W., Nathanielsz P. W. and Du M. (2006). Maternal nutrient restriction affects properties of skeletal muscle in offspring. *J. Physiol.* 575, 241-250. 10.1113/jphysiol.2006.112110 16763001 PMC1819430

